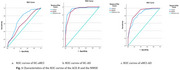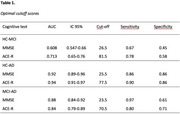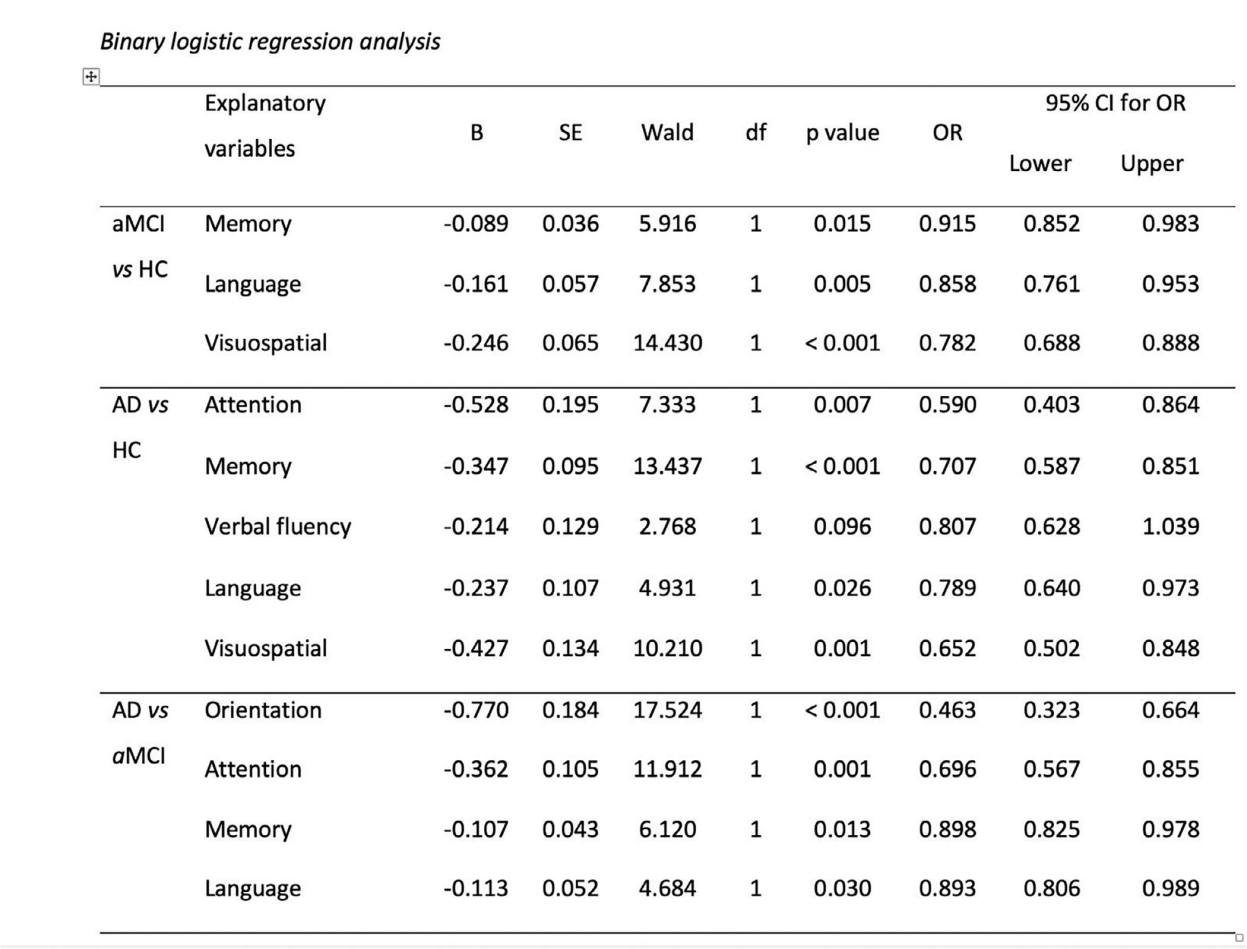# Validity of two cognitive screening markers to distinguish between amnestic mild cognitive impairment (amMCI), Alzheimer's disease (AD) and healthy controls (HC) in low‐income populations with limited educational attainment

**DOI:** 10.1002/alz70856_105617

**Published:** 2026-01-09

**Authors:** Alfredis Gonzalez‐Hernandez, Jasmin Bonilla‐Santos, Dorian Cala, Duvan Gomez, María Fernanda Aguilar Mora, Laura Natalia Calceto Garavito

**Affiliations:** ^1^ Surcolombiana University, Neiva, Huila, Colombia; ^2^ Cooperative University of Colombia, Neiva, Huila, Colombia; ^3^ Universidad Surcolombiana, Neiva, Huila, Colombia

## Abstract

**Background:**

The low levels of educational attainment in Colombia, closely tied to socioeconomic inequalities, contribute to the cognitive and functional decline of older adults (Santamaria‐Garcia et al., 2023). These disparities explain 24.6% to 30% of brain variability linked to neurodegeneration in Latin America, intensifying cognitive decline in vulnerable populations (Gonzalez‐Gomez et al., 2024). Among cognitive screening tools, Addenbrooke's Cognitive Examination‐Revised (ACE‐R) effectively differentiates cognitive disorders. This study aimed to (a) evaluate the reliability and validity of two screening markers distinguishing amnestic mild cognitive impairment (aMCI), Alzheimer's disease (AD), and healthy controls (HC) in low‐income populations with limited education, and (b) examine the contribution of ACE‐R subdomains as explanatory variables for diagnosing aMCI and AD, while accounting for demographic factors like age, sex, and education.

**Methods:**

The sample included 421 participants: 129 HC, 216 individuals with aMCI, and 76 individuals with AD. Diagnosis of aMCI was based on Petersen criteria (Petersen et al., 2014) and Winblad criteria (Winblad et al., 2004), while dementia diagnoses followed NINCDS‐ADRDA guidelines (McKhann et al., 1984). The average schooling of the sample was 5.5 (3.9), and the age was 65.5 (7.7).

**Results:**

The ACE‐R test can be used as a diagnostic tool, to establish the diagnosis of aMCI vs HC, and aMCI vs controls. ROC analysis demonstrated that the ACE‐R achieved an AUC of 0.71, compared to 0.60 for the MMSE, in differentiating aMCI from HC. In distinguishing HC from AD, the AUC was 0.94 for the ACE‐R and 0.92 for the MMSE. Binary logistic regression analysis using backward stepwise selection (with likelihood ratio) identified significant predictors for differentiating aMCI from HC, including memory (OR=0.91; CI 95%: 0.85–0.98), language (OR=0.85; CI: 0.76–0.95), and visuospatial abilities (OR=0.78; CI: 0.68–0.88). Similarly, AD could be differentiated from aMCI based on orientation (OR=0.46; CI: 0.32–0.66), attention (OR=0.69; CI: 0.56–0.85), memory (OR=0.89; CI: 0.82–0.97), and language (OR=0.89; CI: 0.80–0.98).

**Conclusion:**

The Colombian ACE‐R demonstrated superior diagnostic performance in low‐income populations with limited education compared to the MMSE. Its subdomains, particularly memory and language, are critical for identifying early cognitive impairment, underscoring the need for culturally adapted tools for underserved populations.